# Evaluation of color changes of white spot lesions treated with three different treatment approaches: an in-vitro study

**DOI:** 10.1590/2177-6709.25.1.27.e1-7.onl

**Published:** 2020

**Authors:** Shaza M. Hammad, Noha A. El-Wassefy, Mohamed A. Alsayed

**Affiliations:** 1 Mansoura University, Faculty of Dentistry, Department of Orthodontics (Mansoura, Egypt).; 2 Mansoura University, Faculty of Dentistry, Department of Dental Biomaterials (Mansoura, Egypt).

**Keywords:** White spot lesions, Resin infiltration, Nano-hydroxyapatite, Microabrasion

## Abstract

**Objective:**

To qualitatively and quantitatively assess the color changes effect and the color stability of the resin infiltrant on white spot lesions (WSLs), in comparison with nano-hydroxyapatite (nano-HA) toothpaste and microabrasion.

**Methods:**

WSLs were artificially created on sixty human premolars enamel surfaces and randomly assigned to equal four groups (n = 15 each): nano-HA toothpaste, microabrasion (Opalusture), resin infiltrant (Icon) treatment, or artificial saliva (control group). The color change (ΔE) of each specimen was measured by dental spectrophotometer (Vita Easyshade) at different time points: baseline, after WSLs’ creation, after application of treatments, one month, three and six months after treatments application.

**Results:**

The ΔE value did not differ significantly for the four groups at baseline measurement before treatment (*p*> 0.05). Icon resin infiltrant improved the color of WSLs significantly immediately after its application, giving the lowest ΔE value (3.00 ± 0.59), when compared to other treatments (*p*< 0.001). There were no significant changes in ΔE (*p*> 0.05) for all groups during the follow up intervals (one month, three and six months after treatments application).

**Conclusion:**

Resin infiltrant can improve the color of WSLs and restore the natural appearance of enamel better than nano-HA toothpaste and microabrasion.

## RESUMO

**Objetivo:**

avaliar qualitativamente e quantitativamente os efeitos nas mudanças e estabilidade da cor de lesões de mancha branca (LMBs), após tratamento com infiltração de resina, em comparação aos tratamentos com pasta de dentes com nanopartículas de hidroxiapatita (Nano-HA) e com microabrasão.

**Métodos:**

As LMBs foram criadas artificialmente em 60 superfícies de esmalte dentário de pré-molares humanos e aleatoriamente divididas em quatro grupos (n=15, cada): pasta de dentes Nano-HA, microabrasão (Opalusture), tratamento com infiltração de resina (Icon) e saliva artificial (grupo controle). A mudança de cor (ΔE) de cada espécime foi aferida com um espectrofotômetro odontológico (Vita Easyshade) em diferentes tempos: início do estudo, após a criação das LMBs, após a aplicação dos tratamentos, um mês, três meses e seis meses após a aplicação dos tratamentos.

**Resultados:**

Os valores de ΔE não diferiram significativamente entre os quatro grupos ao início do estudo (*p* > 0,05). A infiltração com resina Icon melhorou significativamente a coloração das LMBs imediatamente após a sua aplicação, com o menor valor de ΔE (3,00 ± 0,59), quando comparada às outras modalidades de tratamento (*p* < 0,001). Não houve mudanças significativas nos valores de ΔE (*p* > 0,05) em qualquer um dos grupos durante os intervalos de acompanhamento (um mês, três meses e seis meses após a aplicação do tratamento).

**Conclusão:**

A infiltração de resina é capaz de melhorar a coloração das LMBs e restaurar a aparência natural do esmalte de forma superior à pasta de dentes com Nano-HA e à microabrasão.

## INTRODUCTION

Demineralization of enamel around orthodontic brackets is the most prevalent and undesirable complication of fixed orthodontic appliances, affecting nearly 50% of the orthodontic patients.^1^ The main reason for the lesions’ development is the stagnation of plaque around orthodontic brackets, beneath the archwires, and between brackets and gingival margin.^2^ The white spot lesions (WSLs) can appear one month after bonding of fixed appliances and may last for up to five years after de-bonding of the appliances, compromising patients’ esthetics.^3^

WSLs are preventable with proper oral hygiene, which depends mostly on patient compliance. It has been reported that there is a significant association between poor compliance with home care preventive procedures and the formation of WSLs.^4^ Geiger et al.^5^ reported that less than 15% of patients with orthodontic appliances followed the instructions of rinsing their mouths daily. Twice daily tooth brushing is recommended by many clinicians as an essential part of daily plaque control routine for all orthodontic patients.^6^ Although periodic reinforcement by any clinician may help to motivate a patient, keeping a regular and efficient hygiene is reported to be a difficult process.^4^ Professional oral hygiene instructions and regular professional cleaning has been shown to be effective in reducing decalcification, especially when the degree of patients’ compliance is poor.^4^

The treatment of WSLs usually includes non-invasive approaches, such as remineralization, which is the first line of treatment, minimally invasive approaches such as resin infiltration, or invasive approaches such as microabrasion, composite restorations or even more invasive approaches such as veneers or crowns. 

Many reports have shown that nano-hydroxyapatite (nano-HA) is capable of remineralizing artificial carious lesions after its addition to mouthwashes and toothpastes.^7^ ^,^ ^8^ Microabrasion can be also used for the removal of superficial non-carious enamel defects. Some studies advocated its use for the removal of post-orthodontic WSLs.^9^

Resin infiltration technique was introduced into dentistry with the aim of arresting incipient carious lesions’ progression with low-viscosity light-curing resins.^10^ The resin fills the inter-crystalline spaces and pores in the demineralized enamel, which creates a diffusion barrier on the surface of enamel and within its deeper layers, occluding pathways for entry of acids into the enamel, and so stopping progression of the lesions.^11^ Recent studies suggested that resin infiltration can restore the color of WSLs to a clinically acceptable level.^12^ ^,^ ^13^

Up to our knowledge, no study compared the color changes effect and color stability of resin infiltrant, as a minimally invasive treatment approach for WSLs, with nano-HA toothpaste as a non-invasive treatment approach, and microabrasion as an invasive treatment approach, over follow-up time intervals, so this study was conducted.

## MATERIAL AND METHODS

### Sample size

The sample was selected by convenience and the number included in each group was comparable to a previous study by Yuan et al.^12^ (n=13). 

### Teeth selection and specimens’ preparation

Sixty sound human premolars were collected from orthodontic patients, according to the Ethical Committee of Mansoura University approved protocol number (33020418). These teeth were indicated for extraction for orthodontic reasons and were unidentified to which patient they belonged. Immediately after extraction, the teeth were washed and cleaned with distilled deionized water (DDW) to remove any soft tissue debris or blood. The teeth were examined by stereomicroscope (SZ-PT model, Olympus, Japan) at 10 x magnification, to ensure that they were free from: stain, demineralization, hypoplasia, decay, fluorosis, enamel defects or cracks. All teeth with history of exposure to bleaching agents were excluded. The collected teeth were then cleaned using polishing brush and non-fluoride polishing paste (Nupro cups, Dentsply, USA) with a low speed hand-piece (NSK, Japan) under water cooling system. Each tooth was sectioned mesio-distally using a cutting machine (Isomet 4000 microsaw, Buehler, USA). The buccal halves of 60 premolars were utilized in this study. Teeth were then stored in DDW; changed daily, at room temperature until time of use.

### Standardization procedure

To standardize the area of color assessment, an index made from polypropylene polymer transparent sheets (Essix C+, Dentsply, USA) was utilized. Five specimens were mounted in a typodont and a vacuum-forming machine was used to cover them with sheets. The edges of each index were trimmed with a low-speed stone to correspond to the cemento-enamel junction. Each index was labelled with letters (C “control”, M “microabrasion”, N “nano-HA” or I “Icon”) and numbers (1 to 15) to represent the corresponding specimen. Then a round window with 5-mm diameter was opened in the center of the buccal surface of the index with a low-speed carbide stone (Dentsply, York, PA, USA) of the same diameter, to serve as the area of color measurement by the spectrophotometer. The diameter of each opening was checked using a caliper.

### Development of artificial WSLs

A piece of adhesive tape with 5-mm diameter was attached on the buccal surface of each specimen, corresponding to the window created in each index for color measurements. Two layers of colorless acid resistant nail varnish (Maybelline, USA) were consequently painted and left to dry individually at room temperature on each specimen for 24 hours.

Artificial WSLs were created according to the protocol described by Featherstone et al.^14^ The demineralizing solution consisted of 2 mM CaCl_2_, 2 mM NaH_2_PO_4_, and 50 mM CH_3_COOH to adjust the pH to 4.55. The remineralizing solution consisted of 2 mM CaCl_2_, 2 mM NaH_2_PO_4_, with the addition of 0.1M NaOH to obtain a pH of 6.8. The pH of solutions was checked using a pH meter (pH-2011, LIDA, China).

Each specimen was soaked in 40 ml of demineralizing solution in a tightly sealed plastic container^15^ for 6 hours per day, then removed, cleaned with DDW and immersed in another plastic container filled with the remineralizing solution for 18 hours per day for 5 consecutive days. Solutions were refreshed daily. This procedure leads to the formation of artificial lesions that closely resembles to that obtained adjacent to orthodontic brackets.^16^

The specimens were numbered from 1 to 60 and randomly divided by a computer software into four equal groups (n=15) according to the treatment protocol: microabrasion group, nano-HA toothpaste group, resin infiltration group or remain untreated “control group”.

### Color evaluation at baseline and after

#### WSLs creation

Baseline specimens’ color was assessed using a dental spectrophotometer, Vita Easyshade (Vita Zahnfabrik, Bad Säckingen, Germany) before the creation of WSLs. At each time interval in the study, the color was assessed three times for each specimen and the mean value was calculated and recorded. A dark box was used during color measurement to prevent light interference and ensure standardization. After demineralization, the specimens were washed twice with DDW, gently air dried, and then, the specimens’ color was assessed.

#### Preparation of nano-HA toothpaste

Nano-HA powder was prepared using the wet precipitation method. This method was chosen due to its relative easiness and its reliability, which depends on the production of polycrystalline HA loose powder from aqueous solutions.^17^ The size of the prepared particles ranged from 10 to 20 nm. The toothpaste was prepared with a concentration of 10% wt of nano-HA, according to the method described by Laba^18^ using the following ingredients; 2.5% wt carbopols, 10.0 % wt silicon oxide , 40.0 % wt sodium bicarbonate, 2.5 %wt Sodium lauryl sulfate, 35.0% wt glycerin and 10.0 % wt nano-HA powder.

#### Treatments application

For the microabrasion group (n=15), the WSLs surface was microabraded only once for each specimen. The procedure was performed by using equal amounts of Opalustre microabrasion paste on the labial surface of the WSLs and Opal Prophy Cups (Ultradent Products, Inc., South Jordan, UT, USA) with a low speed contra-angle handpiece (NSK, Japan), at 5000 rpm for 60 seconds. A new Prophy Cup was used for each specimen.

For the nano-HA group (n=15), a soft toothbrush (Oral B, USA) was used to brush the labial surface of each specimen with nano-HA toothpaste for 60 seconds two times per day for ten consecutive days.^19^ An approximate equal volume of 2 ml^3^ of the toothpaste was used for each specimen every time.

For the resin infiltration group (n=15), the resin infiltrant (Icon, DMG, Hamburg, Germany) was applied according to the manufacturer’s instructions: 15% HCl (Icon Etch, DMG, Hamburg, Germany) was directly applied from the syringe and left for 2 minutes, then rinsed off with water for 30 seconds. The WSLs were desiccated for 10 seconds using air-water spray, followed by the application of 99% ethanol (Icon Dry, DMG, Hamburg, Germany), and air-dried for 10 seconds. Resin infiltrant (Icon Infiltrant, DMG, Hamburg, Germany) was applied to the WSLs with the sponge applicator provided with the kit, and left for 3 minutes, then light-cured for 40 seconds using LED light curing unit (Elipar S10, 3M ESPE, USA) with an intensity of 1200 mW/cm^2^. Then, a second coat of Infiltrant was applied and left for 1 minute, then light-cured for 40 seconds. To apply standardized amounts of Icon-Etch, Icon-dry and Icon Infiltrant per specimen, each syringe screw was rotated in half a turn upon each application.

After the treatment application, all specimens were washed with DDW, the nail varnish was carefully removed with colorless acetone, and then specimens were again washed with DDW and prepared for color measurement.

#### Spectrophotometric color assessment

The obtained color measurement values were presented using the Commission Internationale de l’Eclairage (CIE) L*a*b*.^20^ The L* axis represents the degree of lightness within a sample and ranges from 0 (black) to 100 (white). The a* value represents the red/green axis, where an increase indicates a higher red color component. The b* value represents the yellow/blue axis, where an increase indicates higher yellow color component.

Readings obtained from spectrophotometer were expressed in the form of CIE L*a*b* color parameters. The total color difference (ΔE) between different time intervals of the study was calculated in relation to baseline sound enamel readings using the following formula:

$$\Delta E\;=\;{\left[{\left(L_{1}-L_{2}\right)}^{2}+\;{\left(a_{1}-a_{2}\right)}^{2}+\;{\left(b_{1}-b_{2}\right)}^{2}\right]}^{1/2}$$

In our study, the color was assessed six times at different six time points for each specimen. Firstly, the color was assessed before WSLs creation on each specimen, to serve as baseline sound enamel. Secondly, the color was assessed after the creation of WSLs. Then, one month, three and six months after the application of treatments.

#### Fluorescence stereoscope

Fluorescence stereoscope (SZX2-ILLK, Olympus, Japan) was used to capture images of selected samples at the same time points. All the procedures were performed by the same operator.

### Statistical analysis

Data were analyzed with IBM SPSS version 21 for windows (SPSS Inc., IBM Corporation, NY, USA). The normality of data was first tested with one-sample Kolmogorov-Smirnov test. Continuous variables were presented as mean ± standard deviation (SD). ANOVA test was used to compare more than two groups while paired t-test was used to compare paired two groups.

## RESULTS

Table 1 shows mean and SD of the color change (ΔE) for the different treatment groups and their baseline color.

**Table 1:** SD of the color change (ΔE) between different treatment groups and their baseline color.

Variables	Control (n=15)	Microabrasion (n=15)	Nano-HA (n=15)	Icon (n=15)	ANOVA test	P- value
WSLs	15.83±2.30	15.41±2.27	16.15±2.48	15.01±1.99	0.713	0.548
0-month	15.79±2.32	11.99±2.16^a^	10.96±1.59^a^	3.00±0.59^abc^	133.538	<0.001**
1-month	15.56±2.14	11.87±2.26^a^	10.61±1.83^a^	2.87±0.51^abc^	128.163	<0.001**
3-months	15.54±1.95	11.75±1.87^a^	10.55±1.83^a^	2.84±0.56^abc^	155.064	<0.001**
6-months	15.33±1.77	11.60±1.68^a^	10.47±1.60^a^	2.84±0.54^abc^	186.583	<0.001**

Data expressed as mean ± SD. ^a^ Significant with Control group p<0.05. ^b^ Significant with Microabrasion group. ^c^ Significant with Nano-HA group.

**Figure 1:**
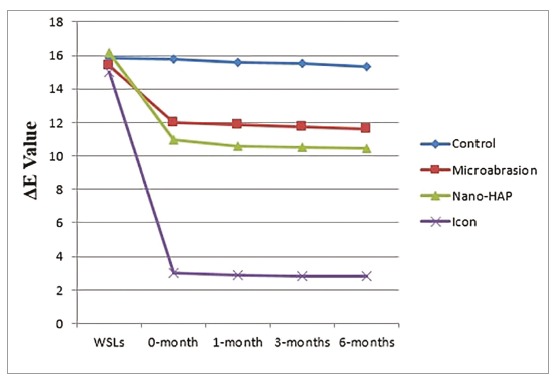
Color changes (ΔE) of artificial WSLs of the four groups at different time points.

The color change (ΔE) did not differ significantly between the control group (15.83±2.30), the microabrasion group (15.41±2.27), the nano-HA group (16.15±2.48) and the Icon group (15.01±1.99) at the baseline color measurement and after completion of WSLs formation (*p*> 0.05). After treatment application (0-month), the color of WSLs in microabrasion group was improved to ΔE=11.99±2.16, the nano-HA group was improved to ΔE=10.96±1.59. Both groups showed significant difference to control group (ΔE=15.79±2.32) (*p*< 0.001), while there was no significant difference between the two groups (*p*> 0.05). The Icon group showed the lowest mean of color change (ΔE=3.00±0.59) that was significantly higher than the other three groups (*p*< 0.001).There was no significant difference in ΔE for all the groups during the follow up intervals (one month, three and six months) after treatments application (*p*> 0.05) (Fig. 1).

### Fluorescence stereoscope

**Figure 2:**
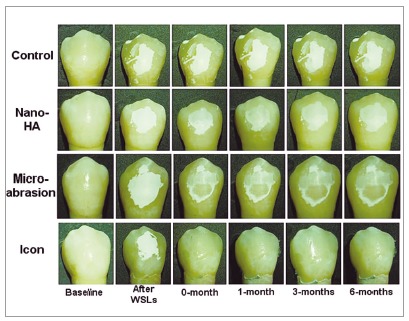
Color change of white spot lesions exposed to different treatments at different time points.

**Figure 3:**
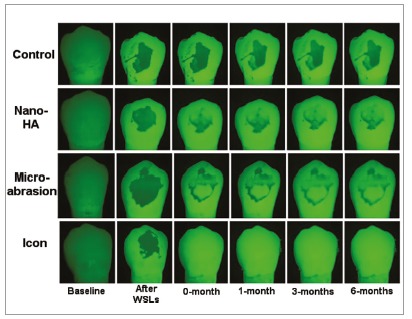
Fluorescence change of white spot lesions exposed to different treatments at different time points.

Selected samples from the four groups were used to capture images under both white light and fluorescence at the same time points of color assessment (Figs 2 and 3). Both figures show a significant and dramatic improvement in the appearance of the WSLs in the Icon group under white light (Fig 2) and fluorescence (Fig 3), when compared to the other three groups immediately after treatments application, and this improvement was stable during the follow-up time intervals.

## DISCUSSION

This study was conducted to qualitatively and quantitatively assess the color changes effect and the color stability of the resin infiltrant on WSLs, in comparison with nano-HA toothpaste and microabrasion, over different time points (0 month, 1 month, 3 and 6 months). Significant decrease in ΔE value in Icon group was noted, in comparison to microabrasion and nano-HA toothpaste groups, indicating that Icon was the most effective treatment for masking the whitish appearance of WSLs. Because an average of 3.3 ΔE was reported to be esthetically acceptable, and that any difference above this limit is highly perceptible and clinically unacceptable,^21^ an average of 3.00±0.59 ΔE obtained with Icon group indicated a better color improvement and acceptable appearance, compared with microabrasion (11.99±2.16) and nano-HA toothpaste (10.96±1.59) groups.

Demineralized enamel surface appears whitish in color due to the difference in refractive indices (RIs) between defective and sound enamel.^22^ This difference is due to the presence of micro-porosities in affected enamel lesions. These micro-porosities are filled with either water (RI=1.33) or air (RI=1.0), unlike sound enamel, which has a RI of 1.62. When these pores are filled with water, the lesions appear opaque compared to sound tissue, while when they are dried they become filled with air and the lesion becomes more apparent and obvious. Thus, the difference in color is believed to be due to the difference in RIs between enamel crystals and medium inside the porosities, which causes light to scatter, resulting in a whitish opaque appearance of those lesions, especially when they are desiccated.^23^ When the low viscosity resin infiltrates these micro-porosities, which were earlier filled with water (RI=1.33), they became now filled with resin infiltrant (RI=1.46), whose RI matches more closely that of enamel (1.62).^24^ The difference in RIs drops to a negligible level and the lesions lose their whitish appearance and blend well with the surrounding sound enamel, expressing a more translucent natural appearance.^25^ The stability of resin infiltrant was explained by its properties of low viscosity, low contact angle and high penetration index, which enables it to penetrate into the deeper layers of the body of WSLs, leading to almost complete plugging of the porosities within WSLs. This plugging reduces the scattering of the reflected light, so improves the color by having a more close light RI.

Nano-HA was shown to deposit on a demineralized surface and to form a new homogenous apatite surface layer, which protects the underlying diseased surface from further demineralization and promotes remineralization.^26^ Nevertheless, nano-HA helps more minerals to deposit on the outer layer rather than the body of the lesion, and then diffusion of mineral ions into deep regions of a lesion can be inhibited by this new highly mineralized surface layer.^27^ This deposition causes the development of the highly mineralized external layer, which is hypothesized to reduce demineralization progress, inhibiting diffusion of acids into deeper areas of enamel, but also may block diffusion of mineral ions into the lesion body, thus constraining the enamel recrystallization to the subsurface zone.^28^ This may explain why complete remineralization, and hence complete color improvement of WSLs was not achieved by the application of nano-HA toothpaste.

During the microabrasion procedure, mild surface abrasion of the enamel prisms with simultaneous acid erosion takes place, compacting mineralized tissue with the newly exposed highly polished surface layer, which replaces the outer prism-free layer. The improvement in esthetics of WSLs may be attributed to light reflection through this new surface, which is thought to act differently than light reflected from an untreated enamel surface.^29^ However, to obtain this improvement, a relatively important layer of enamel has to be removed, and since the depth of WSLs can reach one third of the enamel thickness, this may not be accepted, because modern dentistry aims at maximal tissue preservation. Also, some studies showed that even if up to 360 µm are removed from the enamel surface during microabrasion, the WSL may still be visible in the end.^23^ However, the clinically unacceptable color improvement obtained for the microabrasion group may be explained by the single application of the microabrasion, and repeated applications may be required for better color improvement in clinical practice, though this might increase the amount of sacrificed enamel.

Only short term studies about the stability of the color improvement obtained by the resin infiltrant on post-orthodontic WSLs were realized until now,^30^ but what will happen with this improvement after many years under more challenging discoloring oral environment is still unclear, so further long-term studies are recommended to explain this behavior.

## CONCLUSION

Resin infiltration can improve the color of WSLs and restore natural appearance of enamel. This improvement is stable for relatively long periods; however, long-term evaluation of its stability in clinical practice is needed.
